# Crystallographic and Cellular Characterisation of Two Mechanisms Stabilising the Native Fold of α_1_-Antitrypsin: Implications for Disease and Drug Design

**DOI:** 10.1016/j.jmb.2009.01.069

**Published:** 2009-04-10

**Authors:** Bibek Gooptu, Elena Miranda, Irene Nobeli, Meera Mallya, Andrew Purkiss, Sarah C. Leigh Brown, Charlotte Summers, Russell L. Phillips, David A. Lomas, Tracey E. Barrett

**Affiliations:** 1 School of Crystallography, Birkbeck College, University of London, Malet Street, London WC1E 7HX, UK; 2 Department of Medicine, University of Cambridge, Cambridge Institute for Medical Research, Wellcome Trust/MRC Building, Hills Road, Cambridge CB2 0XY, UK

**Keywords:** serpin, *ser*ine *p*rotease *in*hibitor, PDB, Protein Data Bank, NCS, noncrystallographic symmetry, TUG, transverse urea gradient, PEG, polyethylene glycol, serpin, α_1_-antitrypsin, polymerisation, rational drug design, conformational disease

## Abstract

The common Z mutant (Glu342Lys) of α_1_-antitrypsin results in the formation of polymers that are retained within hepatocytes. This causes liver disease whilst the plasma deficiency of an important proteinase inhibitor predisposes to emphysema. The Thr114Phe and Gly117Phe mutations border a surface cavity identified as a target for rational drug design. These mutations preserve inhibitory activity but reduce the polymerisation of wild-type native α_1_-antitrypsin *in vitro* and increase secretion in a *Xenopus* oocyte model of disease. To understand these effects, we have crystallised both mutants and solved their structures. The 2.2 Å structure of Thr114Phe α_1_-antitrypsin demonstrates that the effects of the mutation are mediated entirely by well-defined partial cavity blockade and allows *in silico* screening of fragments capable of mimicking the effects of the mutation. The Gly117Phe mutation operates differently, repacking aromatic side chains in the helix F–β-sheet A interface to induce a half-turn downward shift of the adjacent F helix. We have further characterised the effects of these two mutations in combination with the Z mutation in a eukaryotic cell model of disease. Both mutations increase the secretion of Z α_1_-antitrypsin in the native conformation, but the double mutants remain more polymerogenic than the wild-type (M) protein. Taken together, these data support different mechanisms by which the Thr114Phe and Gly117Phe mutations stabilise the native fold of α_1_-antitrypsin and increase secretion of monomeric protein in cell models of disease.

## Introduction

The plasma protein α_1_-antitrypsin is principally synthesised by hepatocytes and protects lung tissue from degradation by inhibiting neutrophil elastase.^1^ It is the archetypal member of the serpin (*ser*ine *p*rotease *in*hibitor) superfamily of proteins.^2^ In common with other inhibitory serpins, the native fold of α_1_-antitrypsin is metastable and this property is utilised in the characteristic serpin mechanism of action.^3^ The reactive loop of the molecule is oriented into solution as an ideal substrate for its target protease. Following docking, elastase cleaves the P1–P1′^4^ bond of α_1_-antitrypsin, allowing the P1–P14 residues of the reactive loop to insert as an additional strand within β-sheet A. The enzyme remains bound to the P1 residue in a catalytic intermediate state and is translocated 70 Å from one pole of the molecule to the other.^5^ The energy released by the formation of the new β-strand disables the catalytic site and stabilises the covalently bound complex, which is then rapidly cleared from the circulation.^6^

This dynamic mechanism renders α_1_-antitrypsin vulnerable to point mutations, predisposing it to alternative, pathogenic, conformational transitions—in particular self-association into polymer chains.^7^ These accumulate within the endoplasmic reticulum of hepatocytes rather than enter the circulation. The most clinically significant of these is the Z mutation (Glu342Lys) carried by 1 in 27 of North European populations. Homozygotes for the Z allele have deficiency of circulating α_1_-antitrypsin with levels that are 10–15% of those found in homozygotes for M (wild type) α_1_-antitrypsin. Disease ensues from both the toxic effects of polymers aggregated within hepatocytes (neonatal hepatitis, cirrhosis and hepatocellular carcinoma) and the loss of antiprotease activity within the lung (emphysema).

Over the last 15 years, biochemical, biophysical and structural studies have defined the general scheme of α_1_-antitrypsin polymerisation.^7–11^ This proceeds via an initial monomeric intermediate termed M⁎ in a conformation that is activated for the formation of polymers. The existence of this initiating species is indicated by fluorescence studies of α_1_-antitrypsin polymerisation kinetics and by the structure of the thermodynamically stabilised yet highly polymerogenic δ conformer of the closely related protein, α_1_-antichymotrypsin.^8–12^ These studies support the hypothesis that the native-to-M⁎ transition results from dysregulated opening of β-sheet A and allows the reactive site loop of a neighbouring molecule to insert aberrantly as an extra β-strand. Successive intermolecular loop–sheet interactions lengthen the polymer chain. Recently, an alternative model has been proposed, based upon the structure of a self-terminating dimer of the serpin antithrombin,^13^ emphasising polymerisation as an alternative endpoint on the folding pathway. The repeating subunit of the proposed polymer is considerably unfolded compared to the native structure with intermolecular linkage occurring via a β-hairpin formed from the reactive loop and a contiguous β-strand.

Both proposed mechanisms of polymerisation share key characteristics that can be exploited for the purposes of rational drug design. In both cases, increasing stability of the native fold will reduce the population of the unstable intermediate. Moreover, in both cases, polymerisation requires expansion of β-sheet A and the formation of a six-stranded sheet. For α_1_-antitrypsin, this involves the sealing of a solvent-accessible cavity flanking β-sheet A.^14^ This, therefore, represents an attractive target for rational drug design as small molecules binding here will impede expansion of β-sheet A and, thus, prevent polymerisation by an allosteric mechanism. Evidence for this comes from the effects of mutating either of two residues bordering the cavity—Thr114 and Gly117—to phenylalanine. Both mutations reduce polymerisation of α_1_-antitrypsin by an order of magnitude *in vitro* whilst leaving inhibitory activity unaffected.^14^ Moreover, both mutations partially rescue the defect in the total levels of secreted Z α_1_-antitrypsin in a eukaryotic cell model of disease. The importance of this cavity is highlighted by *in silico* screens that have identified lead compounds that could fill the cavity and, hence, reduce polymerisation.^15^ However, unlike the mutations, all compounds that have proven effective *in vitro* have also abolished inhibitory activity.

To understand the structural mechanisms by which the effects of the Thr114Phe and Gly117Phe mutations are conferred, we have crystallised both α_1_-antitrypsin variants and solved their structures to 2.2 and 3.2 Å, resolution, respectively. The structure of Thr114Phe α_1_-antitrypsin allows us to define a novel target for pharmacophores to block polymer formation whilst preserving inhibitory activity. The structure of the Gly117Phe mutant shows that this mutation exerts its effects by changes in both local packing and the position of the F helix. We have gone on to demonstrate that the two mutations ameliorate the defect in folding of Z α_1_-antitrypsin to the native, functional conformation in a COS-7 cell secretion model. These features extend our understanding of the balance between fold stability and metastability in α_1_-antitrypsin. The data are consistent with both proposed β-sheet-A-linked mechanisms of polymerisation. However, in the case of the single-strand linkage model, they can be integrated with previous findings to propose a coordinated mechanism of β-sheet A expansion from the native monomeric protein.

## Results and Discussion

### Effects of the Thr114Phe mutation arise from partial cavity blockade

**Fig. 1:**
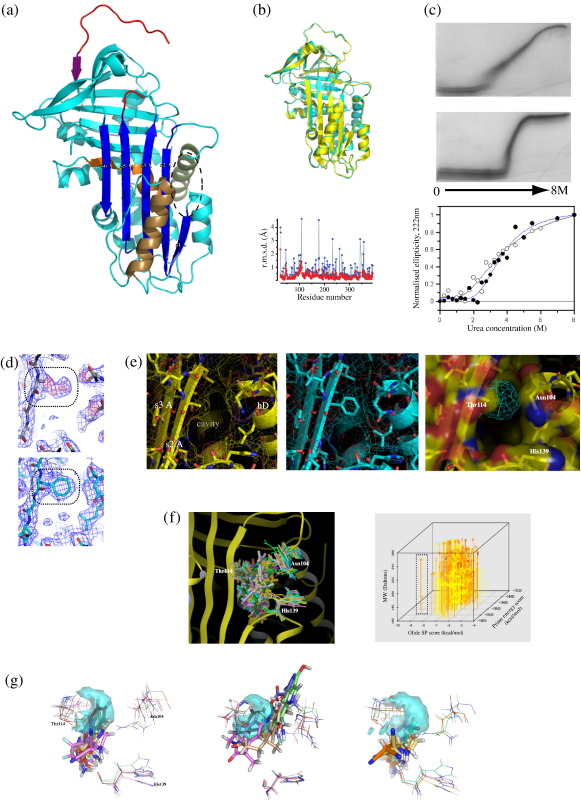
Structure of Thr114Phe α_1_-antitrypsin and *in silico* fragment screen. (a) Crystal structure of Thr114Phe α_1_-antitrypsin shown in cyan with key structural features coloured. The reactive loop is depicted in red, β-sheet A in dark blue, the shutter region in orange, helix D in green, helix F in bronze and strand 1 of β-sheet C (s1C) in purple. The cavity flanking β-sheet A is indicated (ellipse). (b) Superposition of wild-type (PDB code: 1QLP,^16^ yellow) and Thr114Phe (cyan) α_1_-antitrypsin with plot of the r.m.s.d. between the main chains (red) and side chains (blue). (c) Increased stability of the native fold in the presence of the Thr114Phe mutation. TUG-PAGE of wild-type (upper panel) and Thr114Phe (middle panel) α_1_-antitrypsin indicates that the mutation confers increased stability of the native fold to increasing urea concentration. CD spectroscopy allows urea stability to be compared more directly. Ellipticity values measured at 222 nm with varying urea concentrations are shown for wild-type and Thr114Phe α_1_-antitrypsin (*n* = 3). Values are normalised to allow direct comparison of the loss of initial signal. Curves were fitted in Grafit 3.0 (Erithacus Software Ltd.) using an equation modelling a simple two-state denaturation as described previously.^8^ Unfolding measured by this method commenced at 0.7 M urea for the wild-type protein (black curve, open circles) and 1.2 M urea for Thr114Phe α_1_-antitrypsin (blue curve, filled circles). The transition midpoints (assuming complete unfolding at 8 M urea) were reached at similar points (3.4 M urea for wild-type α_1_-antitrypsin and 3.5 M for Thr114Phe α_1_-antitrypsin) in both profiles. (d) Electron density for the Thr114Phe mutation site (boxed) prior to restrained refinement (upper panel). The 2.0-Å structure of wild-type α_1_-antitrypsin (1QLP with the mutation site modelled as an alanine was used as the search model, giving the 2*F*_o_ − *F*_c_ electron density map shown in dark blue (contoured at 1 σ). Positive density for the phenylalanine residue appears in the *F*_o_ − *F*_c_ difference map shown in red (contoured at 3 σ). The same site is shown in the refined structure (lower panel). (e) The surface-accessible cavity flanking β-sheet A, shown in close up for wild-type (left panel, yellow) and in the refined structure of Thr114Phe (middle panel, cyan) α_1_-antitrypsin with side chains and electrostatic surfaces. The volume occupied by the Thr114Phe mutation within the cavity defines a pharmacophore target for screening of fragment compounds to mimic its effects [right panel, cyan mesh, within the wild-type α_1_-antitrypsin cavity (transparent surface representation)]. (f) Results of proof-of-principle fragment screen. Left panel: Ensemble of the 65 highest-scoring ligands successfully docked using the induced fit protocol. Right panel: 4-D plot showing characteristics of these ligands (results shown correspond to 903 poses). All are highly drug-like and favourable for drug development according to the Rule of 5^17^ and the Rule of 3.^18^ Heat map colouring indicates ligand–target centroid proximity (≥ 5.0 Å, yellow; 2.5–5.0 Å, orange; ≤ 2.5 Å, red). Glide docking score values < − 6.8 kcal/mol correspond to micromolar binding constants, and values below − 8.2 kcal/mol are consistent with predicted submicromolar *K*_d_. Closer proximity to the pharmacophore target site indicates increased likelihood of a docked moiety mimicking effects of the Thr114Phe mutation. Smaller ligands allow more extensive structural optimisation during drug design.^19^ Four ligands that formed an outlier group in terms of particularly scoring using both Glide SP and Prime scoring algorithms are indicated (box). (g) Examples of ligands with favourable characteristics following induced fit docking. The pharmacophore target is represented as a transparent cyan surface, and ‘receptor residues’ (Asn104, Thr114 and His139) are shown as optimised for each ligand. Ligand colours correspond to those of receptor site residues optimised for their binding. Non-carbon atoms are individually coloured (oxygen, red; nitrogen, dark blue; fluorine, light blue; sulfur, orange). Left panel: the five ligands top-ranked by Glide SP docking score. Middle panel: the five ligands top-ranked by proximity of ligand to the pharmacophore target. Right panel: the ‘favourable outlier group’ defined in (f).

The 2.2 Å crystal structure of Thr114Phe α_1_-antitrypsin adopts the classic structure of a serpin fold in the native conformation (Fig. 1a and Table 1). The polypeptide chain spanning residues 24–393 is highly ordered with the exception of residues 344–351. These comprise the P15–P8 section of the reactive loop, a section that is disordered or associated with high *B*-factors in other crystal structures of metastable serpins.^9,16,20–22^ The key P1 methionine residue that defines the inhibitory specificity of a serpin adopts a canonical conformation.

Comparison of the structure of Thr114Phe α_1_-antitrypsin with the 2.0-Å structure of wild-type α_1_-antitrypsin that is also in the native conformation [Protein Data Bank (PDB) code: 1QLP]^16^ shows a high degree of overall similarity between the two proteins (Fig. 1b). The root-mean-square deviation (r.m.s.d.) of the mutant and wild-type structures, based on C^α^ positions, is 0.4 Å. A number of critical regulators of conformational change in α_1_-antitrypsin have previously been characterised. Apart from the cavity flanking β-sheet A, these are the proximal hinge of the reactive loop,^23^ the arginine pocket stabilising the loop at the P5 position,^24^ interstrand spacing in β-sheet A,^25^ strand 1 of β-sheet C,^26^ the F helix^9,12,27,28^ and the shutter region^29^ (Fig. 1a). These are essentially unchanged in the mutant structure compared to that of wild-type α_1_-antitrypsin.

The lack of perturbation elsewhere in the overall structure of Thr114Phe α_1_-antitrypsin implies that the mutation must be mediating its effect on the fold stability of native α_1_-antitrypsin (Fig. 1c) from within the cavity flanking β-sheet A. The cavity is well defined in this high-resolution structure, permitting interpretation of the effect of the mutation on cavity filling at the side-chain level. The precise location of the aromatic side chain of Phe114 was strongly indicated by the initial density seen within the cavity adjacent to residue 114 when this was modelled as an alanine (to minimise model bias) prior to any restrained refinement (Fig. 1d). Phe114 makes no interactions with nearby residues, and therefore, its effects are entirely due to partial filling of the cavity and changes in its packing environment during unfolding and polymerisation. The aromatic group of Phe114 closes the upper part of the cavity (Fig. 1d) with its side chain just 3.7 Å from Leu103 and 4.0 Å from Asn104 at the top of helix D, distances that permit favourable van der Waals contacts. These data support the importance of β-sheet A compaction in stabilising the native conformer against initial unfolding.

### Proof-of-principle *in silico* fragment screening based upon the Thr114Phe mutation

The volume partially filling the cavity in Thr114Phe α_1_-antitrypsin (Fig. 1e) is in a region of high-solvent accessibility in the wild-type protein and also yields the therapeutically desirable combination of effective blockade of polymerisation whilst maintaining inhibitory function. Furthermore, it does not alter the native α_1_-antitrypsin structure beyond the cavity in any way. It is, therefore, attractive to consider it a refined target or an ideal pharmacophore volume, depicted in Fig. 1e as the difference between surface representations of wild-type and Thr114Phe α_1_-antitrypsin around residue 114. The estimated size of this ideal pharmacophore is 59 Å^3^, equivalent to 22% of the total cavity volume in wild-type α_1_-antitrypsin.^16^ A similar reduction in the size of lead compounds compared to those identified by screening against the entire cavity would improve the probability of success in rational drug design.^18^ Lipinski's ‘Rule of 5’^17^ observes that success in drug development is highly correlated with the following: *M*_r_ < 500 Da, lipophilic index (log*P*) < 5, ≤ 5 hydrogen-bond donors, ≤ 10 hydrogen-bond acceptors. A modified version of this, the ‘Rule of 3’, has been proposed to guide drug design via exploration of a greater diversity of chemical space using still smaller (< 300 Da) molecular fragments to identify potential ‘hits’.^19^ The mean change in *M*_r_ in development from lead compound to drug is + 69 Da, and most effective drugs used in clinical practice are 250–320 Da.^19^ Currently, the lead compounds designed to block the entire cavity and shown to be effective *in vitro* are at the upper end of the desirable drug size (310–650 Da).^15^

To consider the region occupied by the Thr114Phe mutation a meaningful pharmacophore target, it must generate hits *in silico* that are plausible in terms of mimicking the mutation's partial cavity blockade, their predicted binding affinities (*K*_d_) and ‘drug-likeness’. The pharmacophore target is centred between the middle of strand 2 of β-sheet A (s2A) and the top of helix D (Fig. 1f). It is close to polar residues (Asn104, Thr114 and His139) and an area favourable for hydrophobic contacts that may aid ligand coordination and binding, respectively. On the other hand, unlike the entire cavity, this region is only bounded by residues in one plane, which may be challenging for some docking strategies. These considerations make it hard to assume *a priori* that plausible docking candidates would emerge from *in silico* screening of fragment libraries.

We have therefore performed a proof-of-concept screen of > 70,000 fragments with *M*_r_ = 150–250 Da to assess the potential of the new pharmacophore for identifying small compounds that can be developed in rational drug design. The pharmacophore centroid was set as the midpoint between Thr114 (mutation site, middle of s2A) and Asn104 (top of helix D) for the purposes of our protocol. Screens were performed to select hits on the basis of docking scores and ligand–target centroid proximity. By definition, all hits obey the Rule of 5 and can only deviate from the Rule of 3 a single hydrogen-bond acceptor. The best-scoring 0.1% of ligands were taken forward for induced fit docking, resulting in optimised docking of 65 unique ligands. These had the following mean (± SD) characteristics: *M*_r_ = 207.2 Da (± 21.7), estimated log*P* value = 1.76 (± 0.81) and ligand–pharmacophore centroid proximity = 5.16 Å (± 0.76). The fragments are shown docked ensemble in relation to residues 104, 114 and 139 (Fig. 1f, left). The best docking scores and proximity to the pharmacophore centroid and sizes of all ligands docked by the induced fitting protocol are also shown (Fig. 1f, right). Docking scores calculated by Glide SP correlate with predicted *K*_d_ values such that a value < − 6.8 kcal/mol correlates with a predicted micromolar *K*_d_ and a value < − 8.2 kcal/mol correlates with submicromolar affinity. Figure 1f illustrates that this screen identified numerous fragment hits achieving scores within this range. Examples of docked ligands ranked highly by docking scores and fragment–target centroid proximity are shown in Fig. 1g.

Optimisation of this strategy is likely to require screening 1–2 orders of magnitude more fragments and ‘growing’ (up to *M*_r_ ∼ 250 Da) of the best hits to optimise specificity and drug-likeness ahead of *in vitro* testing. However, the hits identified by this preliminary screen are good candidates in terms of size, predicted binding affinities and drug-likeness for further development. Moreover, our screen also identified compounds that docked well to sites above the pharmacophore target site or interacted with the front of β-sheet A (Fig. 1f). Similar hits will aid the ‘growing’ of compounds for future *in vitro* testing.

### The Gly117Phe mutation alters aromatic ring packing in the interface of β-sheet A with the F-helix interface and induces a half-turn downward shift in F-helix position

**Fig. 2:**
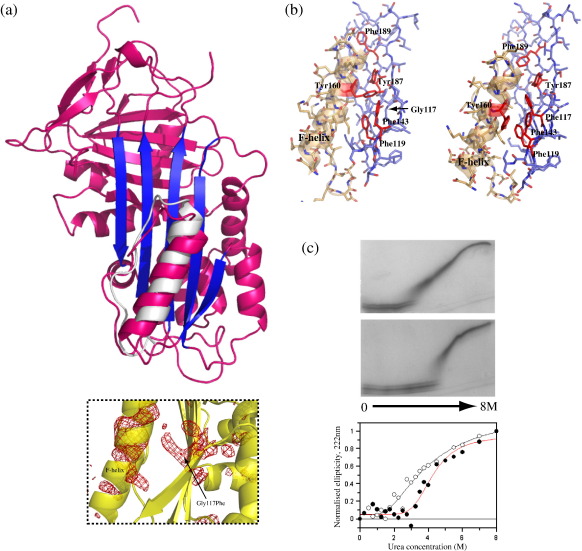
The 3.2-Å structure of Gly117Phe α_1_-antitrypsin. (a) Crystal structure of Gly117Phe α_1_-antitrypsin (pink; β-sheet A in dark blue). The helix F–β-sheet A linker region of the superposed native wild-type protein is shown (white). Box: Initial observation of changes around the mutation site following molecular replacement and rigid-body fitting using the wild-type α_1_-antitrypsin search model (1QLP, shown in yellow). At this stage, difference density (shown in red mesh, contoured at 3 σ) for the mutation site and overlying F helix was calculated by excluding residues 117 and 160 (+ surrounding 3.5 Å spheres) from a simulated annealing omit map to minimise model bias. Similar difference density was seen in the F-helix region in all three copies of the molecule; the example of a single copy is shown. (b) Side-chain packing of aromatic side chains in the helix F–β-sheet A interface in wild-type (left) and Gly117Phe (right) α_1_-antitrypsin. Only β-sheet A (blue, stick representation) and helix F together with its linker region (gold, transparent cartoon and stick representation) are shown. Residues involved in aromatic ring interactions are coloured red, and the mutation site at Gly117 is indicated for wild-type α_1_-antitrypsin. (d) Increased biochemical stability of the native fold in the presence of the Gly117Phe mutation. TUG-PAGE of wild-type (upper panel) and Gly117Phe (middle panel) α_1_-antitrypsin indicates that the mutation confers increased stability of the native fold to increasing urea concentration. This is confirmed by CD spectroscopy (lower panel). For Gly117Phe α_1_-antitrypsin (red curve, filled circles), unfolding begins at 2.0 M urea (compared with 0.7 M for the wild-type protein, black curve, open circles) and the transition midpoint occurs at 4.3 M urea (compared with 3.4 M for wild-type α_1_-antitrypsin). Conditions are as described for Fig. 1c. Taken together, the urea unfolding studies indicate increasing stability of the native fold in the order wild-type < Thr114Phe < Gly117Phe α_1_-antitrypsin.

The structure of Gly117Phe α_1_-antitrypsin was solved to 3.2 Å resolution (Table 1 and Fig. 2a), allowing confident interpretation of the secondary structure of the protein. The asymmetric unit contained three molecules with minimal intermolecular contacts and in an arrangement not suggestive of biologically significant multimerisation. No significant changes are evident in β-sheet A, the shutter region or strand 1 of β-sheet C in Gly117Phe α_1_-antitrypsin relative to the wild-type protein. Electron density was not seen for residues 349–356 (copy A), 348–356 (copy B) and 348–358 (copy C) of the hypermobile reactive site loop.

Before incorporation of the Phe mutation into the model or any restrained refinement had been performed, clear density was apparent at the mutation site, indicating that its side chain was oriented away from β-sheet A towards the F helix where significant changes were seen. To investigate these changes, we omitted the mutation site and the overlying part of the F helix to generate a difference density map of these areas with model bias minimised. This was based upon the initial rigid-body fit of the search model and did not use noncrystallographic symmetry (NCS) restraints, yet it confirmed a half-turn shift of the F-helix along its axis for all three molecules as well as side-chain density for the underlying Phe117 residue (Fig. 2a, boxed). In the refined structure, the F-helix is displaced downwards (towards the bottom of s3A) by half a turn relative to its position in the structure of native wild-type α_1_-antitrypsin (2.7–3.5 Å between equivalent C^α^ atoms). This compares with an overall C^α^ r.m.s.d. of 1.2 Å for the rest of the two structures. The F-helix shift is unlikely to be an artefact of crystal packing as different copies of Gly117Phe α_1_-antitrypsin have different packing environments. Moreover, no F-helix changes are required to permit packing of three molecules of the native wild-type protein in orientations identical with those of the three copies of Gly117Phe α_1_-antitrypsin in the asymmetric unit (not shown). Conversely, repacking of helix F–β-sheet A interface residues, in particular Tyr160, is necessary to avoid steric clashes with the adjacent Phe117 side chain and sustain favourable hydrophobic interactions. Figure 2b compares packing of the helix F–β-sheet A interface in wild-type and Gly117Phe α_1_-antitrypsin. In the refined structure of Gly117Phe α_1_-antitrypsin, Phe117 forms the centre of a cluster of aromatic side chains, interacting with Phe119, Phe143 and Tyr187 (all at distances of 3.5–5.5 Å). The side chain of Tyr160 is in a solvent-exposed position in this mutant in contrast to its buried position within the helix F–β-sheet A interface in wild-type α_1_-antitrypsin but remains well placed for aromatic interactions with two residues of this cluster, Phe119 and Phe143. These differences therefore account for the changed conformation of helix F in Gly117Phe α_1_-antitrypsin compared to the wild-type protein. They introduce an extra aromatic ring into the helix F–β-sheet A interface in the mutant to increase favourable packing interactions between strands 1–3 of the β-sheet. These changes do not obviously increase the degree of hydrophobic interaction between the F helix (or its linker to s3A, both shown in gold) and β-sheet A (blue). However, it is striking that the biochemistry of this mutant^14^ resembles that of α_1_-antitrypsin, carrying what can now be regarded as a complementary mutation, Tyr160Trp.^28^ Here, an extra aromatic ring is introduced from the F-helix side into the helix F–β-sheet A interface to project into a site adjacent to Gly117. The Tyr160Trp mutation is associated with a near-identical increase in thermal stability to that seen with the Gly117Phe mutation. Both mutations are also alike in achieving this without any reduction in inhibitory activity. The increased biochemical stability conferred by the Gly117Phe mutation on the native fold of α_1_-antitrypsin (Fig. 2c) is therefore related to increased stabilisation of the F helix. The relative contributions of the alterations in packing of the helix F–β-sheet A interface and its half-turn displacement towards the lower pole of the molecule remain to be determined. Since these effects involve alterations of the final folding of native α_1_-antitrypsin and are mediated via interactions in a hydrophobic interface, they may be hard to mimic via rational drug design. However, the fact that the position of the F helix is affected in the absence of any other significant changes to the overall fold supports the conclusion that this helix forms relatively late in the folding process and independent from the folding of other structural features.

### Effect of Thr114Phe and Gly117Phe mutations on secretion of Z α_1_-antitrypsin from COS-7 cells

**Fig. 3:**
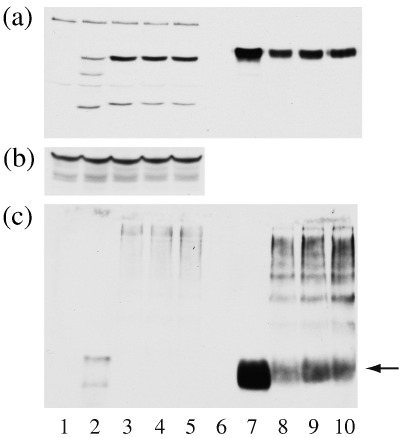
Characterisation of M, Z, Thr114Phe/Z and Gly117Phe/Z α_1_-antitrypsin secreted from COS-7 cells. Western blot analysis of α_1_-antitrypsin retained within or secreted from COS-7 cells 2 days after transient transfection. (a) Western blot analysis of samples run on SDS-PAGE with (b) luciferase transfection control for cell lysate samples [same gel as (a)]. (c) Western blot analysis of samples run on non-denaturing PAGE. Lanes 1–5, cell lysates; lanes 6–10, culture media. Lane 1, empty vector control—cell lysate; lane 2, M α_1_-antitrypsin—cell lysate; lane 3, Z α_1_-antitrypsin—cell lysate; lane 4, Thr114Phe/Z α_1_-antitrypsin—cell lysate; lane 5, Gly117Phe/Z α_1_-antitrypsin—cell lysate; lane 6, empty vector control—supernatant; lane 7, M α_1_-antitrypsin—supernatant; lane 8, Z α_1_-antitrypsin—supernatant; lane 9, Thr114Phe/Z α_1_-antitrypsin—supernatant; lane 10, Gly117Phe/Z α_1_-antitrypsin—supernatant. Arrow indicates migration of native α_1_-antitrypsin secreted into the culture media on non-denaturing PAGE.

To relate our findings to drug design for α_1_-antitrypsin deficiency, we attempted to characterise the stability and folding of the Thr114Phe/Z and Gly117Phe/Z double mutants of α_1_-antitrypsin. These were produced as recombinant proteins using the same overexpression system as the crystallised proteins and purified by the same method. The yield of both double mutants was between 1 and 2 orders of magnitude lower than that of wild-type and single-Phe mutant α_1_-antitrypsin. The products were conformationally heterogeneous with a mixture of native, latent [the major product on non-denaturing and urea polyacrylamide gel electrophoresis (PAGE)] and polymeric conformers. The effects of the Thr114Phe and Gly117Phe mutations on transient expression and secretion of α_1_-antitrypsin containing the Z mutation in a more physiological, eukaryotic cell system were then assessed. Previous work has shown that the Thr114Phe and Gly117Phe mutations partially rescue the profound defect in the overall secretion of α_1_-antitrypsin caused by the Z mutation in a *Xenopus* oocyte system.^14^ These effects are modest in proportion to the overall deficit but are of considerable clinical interest since disease severity correlates closely with rates of polymerisation and (inversely) with circulating levels of α_1_-antitrypsin.^30^ However, this study did not characterise the conformational state of the secreted material. We therefore transformed and transiently expressed M, Z, Thr114Phe/Z and Gly117Phe/Z α_1_-antitrypsin in COS-7 cells. Analysis of cell lysates and culture media by Western blot analysis of sodium dodecyl sulfate (SDS)-PAGE showed increased secretion of α_1_-antitrypsin in the double mutants compared with Z α_1_-antitrypsin (Fig. 3a and b). These data were consistent with those quantified by the more sensitive pulse-chase system in *Xenopus* oocytes. This increase in total α_1_-antitrypsin secretion was related to a clear increase in secretion of native, monomeric α_1_-antitrypsin as assessed by Western blot analysis of non-denaturing PAGE (Fig. 3c, arrow). The absolute concentrations of α_1_-antitrypsin in the culture media were very low relative to those conventionally used to measure the inhibitory activity of α_1_-antitrypsin. However, we were able to detect inhibitory activity using an ELISA technique in which bovine α-chymotrypsin was used to capture functional α_1_-antitrypsin from concentrated culture media. This indicated that more functional material was secreted in the presence of the double mutations (1.35 μg/ml for Thr114Phe/Z and 0.35 μg/ml for Gly117Phe/Z α_1_-antitrypsin) than with the Z mutation alone (< 0.16 μg/ml of active protein; below linear range of detection by ELISA). These effects were modest compared with the amount of functional M α_1_-antitrypsin detected in the culture media (> 5 μg/ml; exceeding the maximum assessable in the linear range of the ELISA) but were consistent with the appearances of native protein by non-denaturing PAGE (Fig. 3c).

The increased secretion of native α_1_-antitrypsin from cells expressing the double mutants compared to those expressing Z α_1_-antitrypsin was also associated with an increase in the amount of polymeric material seen in the media. We cannot formally exclude the possibility that these polymers were formed intracellularly and then secreted differentially. However, the lack of any observable difference in total and polymeric intracellular α_1_-antitrypsin levels between the different constructs does not support this hypothesis. We therefore favour the alternative explanation that the extracellular polymers are formed from α_1_-antitrypsin that is secreted in the native conformation. Taken together, these data support the conclusion that the Thr114Phe and Gly117Phe mutations have modest but detectable effects on increasing secretion of functional native α_1_-antitrypsin on the background of the severe Z mutation in eukaryotic cells. However, these double mutants remain prone to conformational change and polymerisation.

### Relating the crystal structures of Thr114Phe and Gly117Phe α1-antitrypsin to the single- and double-strand linkage models of polymerisation

**Fig. 4:**
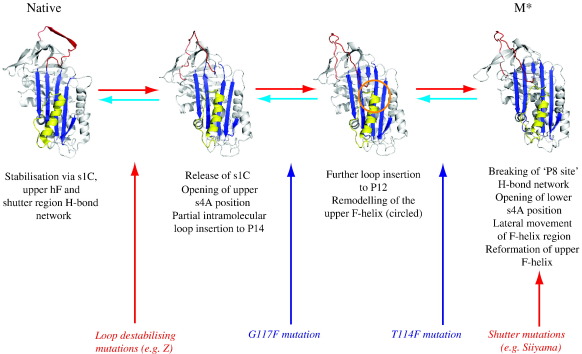
Schema for formation of a partially loop-inserted M⁎ species from native α_1_-antitrypsin. Findings from the Thr114Phe and Gly117Phe α_1_-antitrypsin crystal structures are incorporated together with previous observations of requirements for loss of the s1C^26^ strand, remodeling of the F helix^9,12,28^ and destabilising of interactions involving shutter region residues.^25^ Sequential insertion of the reactive loop into the upper s4A position is depicted by two chimeras. These are derived from structures of native (1QLP) and latent (1IZ2) α_1_-antitrypsin, murine α_1_-antichymotrypsin (1YXA—demonstrating changes associated with opening of the P14 acceptor site) and thyroxine binding globulin (2CEO—demonstrating changes on expansion at the P12 insertion site). In all cases, the reactive loop modelled is that of α_1_-antitrypsin. The final image shows the α_1_-antitrypsin M⁎ model, generated as described in Materials and Methods, after energy minimisation with simulated annealing molecular dynamics. The effects of mutations that will facilitate this transition are shown in red whilst those that block it are shown in blue. Thus, in this scheme, Thr114Phe retards M⁎ formation by cushioning β-sheet A against expansion whilst Gly117Phe stabilises the upper turns of helix F and abolishes the steric requirement for their remodelling in response to partial loop insertion. Conversely, mutations may accelerate polymerisation by favouring partial loop insertion (e.g., Z) or by opening the lower s4A position directly (e.g., Siiyama).

The Thr114Phe and Gly117Phe mutations act by different mechanisms to increase the global stability of native α_1_-antitrypsin. Expansion of β-sheet A is a common feature in both proposed mechanisms of polymerisation.^9,13^ In order for α_1_-antitrypsin to adopt either M⁎ conformation, residue 114 must move laterally by 4.6 Å in the plane of β-sheet A. This is limited by local steric clashes in Thr114Phe α_1_-antitrypsin. In the case of the Gly117Phe mutation, the structural mechanism whereby the changes in the vicinity of the F helix may impede formation of a β-hairpin donor/acceptor M⁎ species is less intuitive. There is no apparent increase in stabilising interactions between the helix F–s3A linker and s5A, and the changes in packing in the interface between the F helix and β-sheet A would not of themselves be expected to impede opening of the β-sheet. Nevertheless, our data are not inconsistent with this model since they may simply reflect overall cooperativity of the final steps on the folding pathway (and initial steps on the unfolding pathway) for α_1_-antitrypsin. However, our findings can be mechanistically integrated with other biochemical data for the native-to-M⁎ transition posited for the single-strand linkage model in which opening of the s4A site is associated with partial intramolecular loop insertion. Various data support the following features of this model: upper s4A opening precedes lower s4A opening^9,25,31,32^ and is associated with partial insertion of the reactive site loop.^9,33,34^ Subsequent lower s4A opening around the site of P8 residue insertion is associated with breaking of a network of interactions between s4A, s5A and shutter region residues.^25^ This can be induced by the insertion of a cleaved reactive loop or a peptide analogue into β-sheet A from the P14–P9 sites, producing species that are highly polymerogenic.^31,32,35,36^ In α_1_-antitrypsin, modelling the insertion of the reactive site loop to the P12 position necessitates both the release of s1C and remodelling of the upper helix F—secondary structural changes known to occur during the formation of polymers from native α_1_-antitrypsin.^12,26^ However, stabilisation of the upper turns of the F helix and/or its downward displacement as seen in the structure of Gly117Phe α_1_-antitrypsin will reduce the propensity for its remodelling. It will, therefore, reduce M⁎ formation if such remodelling is integral to the formation of the intermediate. This process is shown as a schema in Fig. 4. The model of the single-strand donor/acceptor M⁎ is shown following energy minimisation and simulated annealing molecular dynamics (coordinates are supplied as Supplementary Data).

## Conclusion

The data presented here demonstrate in crystallographic detail two mechanisms that greatly stabilise the native fold in α_1_-antitrypsin without abolishing its inhibitory function. These mechanisms are likely to underlie the partial rescue of secretion of native α_1_-antitrypsin when the Thr114Phe or Gly117Phe mutations occur in the context of the Z mutation in eukaryotic cells although the double mutants remain highly polymerogenic. The high-resolution structural characterisation of the Thr114Phe mutation presents a more precise pharmacophore target for screening of small fragments capable of mimicking its effects. These data are generally consistent with either the single-strand or β-hairpin linkage models of polymerisation. However, they suggest a specific mechanism of propagated conformational change in the case of the single-strand model and, therefore, open up new approaches for further studies to test its applicability in this important pathological pathway.

## Materials and Methods

### Site-directed mutagenesis, protein expression, purification and characterisation

QuikChange mutagenesis (Stratagene, California, USA) was used to introduce the Thr114Phe, Gly117Phe and/or Z (Glu342Lys) mutations into His-tagged α_1_-antitrypsin in the pQE31 vector^37^ to create single- or double-mutant constructs. The full-length sequences of the mutated α_1_-antitrypsin genes were confirmed, and plasmids were transformed into XL-1 Blue *Escherichia coli* cells. The wild-type and mutant proteins were expressed and purified as described previously.^14^ The final products were stored in 10 mM Tris/HCl, pH 7.4, 10 mM NaCl and 1 mM ethylenediaminetetraacetic acid. Product purities were confirmed by non-denaturing PAGE, SDS-PAGE and transverse urea gradient (TUG)-PAGE. Further characterisation of urea stability by circular dichroism (CD) was performed as described previously.

The Z, Thr114Phe/Z and Gly117Phe/Z α_1_-antitrypsin constructs for transient transfection of COS-7 cells were prepared by a combination of cloning, subcloning and site-directed mutagenesis in the pcDNA3.1 vector. All constructs were confirmed by sequencing of the whole gene. COS-7 cells were transfected and cultured as described previously.^38^ Samples of the cells in culture media were collected 2 days after transfection. Cells and culture media were separated by centrifugation at 500***g*** for 10 min. Cell lysates and culture media were prepared and assessed by SDS-PAGE and non-denaturing PAGE followed by Western blot analysis for α_1_-antitrypsin as described previously.^38,39^ Inhibitory activity was assessed with an α-chymotrypsin-based ELISA. Each well of a 96-well plate was coated overnight with 100 μl of 5 mg/ml bovine α-chymotrypsin. The plate was then washed before incubating for 15 min at 37 °C with 100 μl of culture medium concentrated 7.5 times (this did not affect relative proportions of polymeric to total α_1_-antitrypsin). After further washing to remove unbound material, the amount of functional material captured was assessed by incubation with 100 μl of purified polyclonal antibody against α_1_-antitrypsin used at 1 μg/ml for 2 h at 4 °C in the presence of 0.25% (w/v) bovine serum albumin as a blocking agent, followed by washing and incubation with 100 μl of horseradish-peroxidase-conjugated anti-rabbit secondary antibody (Sigma-Aldrich, Dorset, UK). The plate was washed and the enzymatic activity was developed with 3,3′,5,5′-tetramethylbenzidine substrate (Sigma-Aldrich). The colour was allowed to develop for approximately 10 min and was halted by the addition of 100 μl of 1 M sulfuric acid to each well. The absorbance was read at 450 nm.

### Crystallisation, data collection and structure solution

Both Thr114Phe and Gly117Phe α_1_-antitrypsin were crystallised by the hanging drop vapour diffusion method in 4-μl drops containing equivalent volumes of 10 mg/ml protein and mother liquor at 20 °C. Thr114Phe α_1_-antitrypsin was crystallised using 0.1 M Li_2_SO_4_, 0.1 M Tris/HCl pH 8.5, and 31% polyethylene glycol (PEG) 4000 (w/v). Gly117Phe α_1_-antitrypsin was crystallised using 0.1 M Li_2_SO_4_, 0.1 M Tris/HCl, pH 8.5, and 25% PEG 4000 (w/v). Crystals were cryoprotected by the addition of 16% PEG 400 (w/v) (for Thr114Phe α_1_-antitrypsin) or 16% ethylene glycol (w/v) (for Gly117Phe α_1_-antitrypsin) before being flash cooled in liquid nitrogen. Diffraction data for Thr114Phe α_1_-antitrypsin were collected at station I03 at the Diamond synchrotron (Oxfordshire, UK), and those for Gly117Phe α_1_-antitrypsin were collected at station ID14.4 at the European Synchrotron Radiation Facility (Grenoble, France). Data were processed using MOSFLM^40^ and scaled using Scala.^41^ Molecular replacement was carried out using MOLREP^42^ with protein coordinates from the 2.0-Å structure of wild-type α_1_-antitrypsin (1QLP)^16^ as the search model. Refinements for both structures were carried out using Refmac^43^ and CNS.^44^ Whilst the asymmetric unit of Thr114Phe α_1_-antitrypsin contained only a single molecule, that of the Gly117Phe mutant contained three molecules. Two molecules were related by a 2-fold rotational symmetry axis, the third was related to the others by translation without an *n*-fold rotational axis. NCS was therefore applied during the solution of the structure of Gly117Phe α_1_-antitrypsin. This was initially applied with all three molecules constrained to be identical. Once *R*_free_ values had converged between successive refinement rounds, the NCS was subsequently applied with weighted restraints. In three regions where the electron density indicated increased intermolecular variability [residues 90–112 (helix D and linker to s2A), 150–180 (helix F and linker to s3A) and 343–363 (reactive site loop)], NCS restraints were not applied. Manual rebuilding of models was performed in Coot,^45^ and figures were produced using PyMOL^46^ and Chimera.^47^

### Characterising the pharmacophore target implied by the Thr114Phe mutation

The cavity flanking β-sheet A in α_1_-antitrypsin was defined and quantified using SURFNET.^48^ As described previously,^16^ grid separations of 0.8 Å and probe spheres of 2.0–4.0 Å radii were used. The extra volume occupied within the cavity by the Thr114Phe mutation was calculated by subtracting the volume of the cavity in the mutant from that in the wild-type protein. Similarly, an ideal pharmacophore capable of mimicking the partial cavity blockade caused by the mutation was illustrated by subtraction of the surface map of residue 114 in wild-type α_1_-antitrypsin from that of Thr114Phe α_1_-antitrypsin using SURFNET.^48^

### *In silico* screens of compound libraries

The fragment-like subset of the ZINC database^49^ (− 2 ≤ xLog*P* ≤ 3, *M*_r_ = 150–250 Da, ≤ 3 rotational bonds, ≤ 2 hydrogen-bond donors and ≤ 4 hydrogen-bond acceptors) was selected for initial prescreening to identify drug- and lead-like compounds capable of significant modification to optimise specificity without unfavourably increasing size. The subset labelled ‘usual’, containing all fragment-like molecules protonated in the pH range 5.75 to 8.25, was downloaded. Programs from the Schrödinger Suite 2007 (Schrödinger LLP, New York, NY, USA) were used for *in silico* docking. Fragments were preprocessed using LigPrep version 2.1. The original ionisation state was retained and ‘desalt’ and ‘tautomer generation’ flag settings were used. Chiralities were determined from the original ZINC database structures, and one low-energy ring conformation was allowed. This procedure resulted in a total of 70,925 small-molecule structures for docking. The protein was prepared using Maestro version 8.0 (Protein Preparation Wizard). Hydrogens were added, and all waters were deleted from the structure of wild-type α_1_-antitrypsin. Hydrogen bonds were optimised, and the structure was minimised within 0.30 Å r.m.s.d. from the initial structure. The potential energy grid was pre-calculated using a box centred on the centroid of residues Asn104 and Thr114 to prepare the binding site for docking. The box was 39 Å in each dimension, but the ligand centre was constrained to lie within 14 Å of the box centre. No additional constraints were used.

Initial screening of the 70,925 ligands was performed using Glide version 4.5, to generate a range of putative ligand–receptor interactions (poses). The best pose for each ligand was calculated using Emodel and retained. Ranking between ligands was then performed using the Glide SP score. All Glide parameters were left to their default values. The top 70 (< 0.1%) ligands resulting from this Glide SP run were then submitted to the Induced Fit Docking protocol, a three-step docking process. Preliminary docking by Glide SP used a scaling factor for van der Waals radii of 0.5 for both the ligand and the receptor. For each ligand, the best 20 poses were retained. Prime version 1.6 was used to optimise receptor side chains within 5.0 Å of each posed ligand. Finally, ligands were redocked into the optimised structures. The resulting poses were ranked using a composite of the Glide SP (binding site energy) and the Prime (molecular mechanics + solvation energy function for the protein–ligand complex) scores as previously described.^50^

The molecular weight and other descriptors for the set of docked ligands were calculated using the MOE suite (MOE 2006.08, Chemical Computing Group, Montreal, QC, Canada). Prior to descriptor calculation, the partial charges on all atoms were recalculated using the default MOE force field (MMFF94x), and adjustment of hydrogens and lone pairs was allowed.

### Modelling α_1_-antitrypsin in the M⁎ conformation

To model wild-type and mutant α_1_-antitrypsin in the partially loop-inserted M⁎ conformation, we used the 2.2-Å crystal structure of latent α_1_-antitrypsin (1QLP)^51^ as the initial template. In the latent conformation, the reactive loop is fully inserted into β-sheet A as its fourth strand (s4A). This structure was chosen as it is the highest-resolution structure of α_1_-antitrypsin with an expanded β-sheet A. Mutations were restored back to the wild-type sequence. The residues of the reactive site loop were threaded into the conformation adopted in the structure of δ Leu55Pro α_1_-antichymotrypsin (1QMN)^9^ using SWISS-MODEL.^52^ As the reactive loop of α_1_-antitrypsin is four residues shorter than that of α_1_-antichymotrypsin, this required stripping out of s1C to connect it directly with s4B. The model was regularised in Coot before undergoing energy minimisation, simulated annealing and final energy minimisation using SANDER, with the protein parameterised using the AMBER99SB force field.^53^ The upper turn of helix F required greater freedom during simulated annealing than the rest of the molecule to allow successful energy minimisation. This was achieved by conservative relaxation of restraints on this region relative to the rest of the molecule.

### Accession numbers

Coordinates and structure factors are deposited in the PDB with accession number 3DRM for the structure of Thr114Phe α_1_-antitrypsin and 3DRU for the structure of Gly117Phe α_1_-antitrypsin.

## Figures and Tables

**Table 1:** X-ray data collection and refinement statistics for structures of Thr114Phe α_1_-antitrypsin and Gly117Phe α_1_-antitrypsin

	Thr114Phe α_1_-antitrypsin	Gly117Phe α_1_-antitrypsin
*Data collection*		
Cell dimensions		
*a* (Å)	113.8	58.8
*b* (Å)	39.6	149.3
*c* (Å)	90.3	77.3
β (°)	105.1	94.11
Molecules in asymmetric unit	1	3
Space group	*C*2	*P*2_1_
Resolution range (Å)	87.0–2.2	77.1–3.2
*R*_sym_ (%)	10.6 (49.3)	13.8 (58.8)
Completeness (%)	94.2 (90.2)	99.7 (99.7)
*I*/σ*I*	15.3 (5.5)	11.5 (2.5)
Multiplicity	6.8 (6.8)	4.7 (4.8)

*Structure refinement*		
No. of atoms (protein)	2847	8562
No. of atoms (water)	164	0
*R*_free_ (%)	27.2	29.0
*R*_cryst_ (%)	22.4	24.2
*B*_ave_		
Main chain	31.8	68.3
Side chain	34.7	74.6
Ramachandran plot statistics (%)		
Favoured regions	89.8	74.6
Allowed regions	9.0	21.7
Generously allowed	1.2	2.8
Disallowed	0.0	0.9
r.m.s.d. from ideality		
Bonds (Å)	0.007	0.010
Angles (°)	1.3	1.6

Values in parentheses refer to the highest-resolution shell. *R*_sym_ = ∑*_i_*(∑*_j_*|∑*I*_*ij*_ − 〈*I*_*i*_〉|)/∑*_i_*〈*I*_*j*_〉, where *j* are the set of observations for each reflection *i*. *R*_cryst_ = ∑*_i_*||*F*_o_| − |*F*_c_||/*F*_o_|. *R*_free_ = *R*_cryst_ for 5% of reflections omitted from refinement. *B*_ave_ values are average temperature factors for all molecules in the asymmetric unit.
